# Poorly differentiated cecal adenocarcinoma showing prominent rhabdoid feature combined with appendiceal mucinous cystadenoma: A case report and review of the literature

**DOI:** 10.3892/ol.2015.2905

**Published:** 2015-01-27

**Authors:** IN-JU CHO, SUNG-SOO KIM, YOUNG-DON MIN, MUN-WHAN NOH, RAN HONG

**Affiliations:** 1 Department of Pathology, College of Medicine, Chosun University, Gwangju 501-759, Republic of Korea; 2 Department of Surgery, College of Medicine, Chosun University, Gwangju 501-759, Republic of Korea; 3 Department of Complementary and Alternative Medicine, Chosun University Graduate School, Gwangju 501-759, Republic of Korea

**Keywords:** rhabdoid, carcinoma

## Abstract

Extrarenal rhabdoid tumors (ERRTs) are extremely rare neoplasms; of these, colorectal ERRTs are the most rare, and only nine cases have been previously described in the English language literature. The current study reports the pathological features of a case of poorly differentiated cecal adenocarcinoma with prominent rhabdoid feature, which was combined with mucinous cystadenoma of the appendix in a 73-year-old male, and additionally reviews the previously reported cases. Microscopically, the majority of tumor cells were non-cohesive or loosely cohesive, with a polygonal morphology and prominent rhabdoid feature, showing eccentric vesicular nuclei, prominent nucleoli and abundant eosinophilic cytoplasm. Immunohistochemically, the tumor cells were positive for cytokeratin (CK) and vimentin, but negative for CK20, CK7, desmin and smooth muscle actin. This indicated a diagnosis of poorly differentiated adenocarcinoma with prominent rhabdoid features, combined with appendiceal mucinous cystadenoma. At two months following surgery the patient succumbed to peritoneal seeding and metastasis of liver and bone The emergence of the rhabdoid phenotype is invariably associated with an aggressive and almost always fatal clinical course. The present case is the 10th example of such a tumor in the colon, and to the best of our knowledge, this is the first case of colonic rhabdoid tumor coinciding with appendiceal benign mucinous neoplasm.

## Introduction

Rhabdoid tumors (RTs) are aggressive neoplasms, initially described by Beckwith and Palmer as a sarcomatoid rhabdoid variant of Wilms’ tumor (1). Tumors with similar clinicopathological characteristics have been subsequently reported in a number of extrarenal sites and associated with an unfavorable prognosis (2). Of these RTs, colorectal cancers with rhabdoid features are extremely rare, and to date, only nine cases have been previously reported (2–9). The most noteworthy morphological feature is the strongly and homogeneously acidophilic cytoplasm of the tumor cells (the result of packing by intermediate filament) with occasional lateral displacement of the nuclei (10). On immunohistochemical analysis, the tumor cells are characteristically positive for vimentin (VMT) and often for cytokeratin (CK) and epithelial membrane antigen (EMA), but generally negative for skeletal muscle marker or S-100 protein (11). Rhabdoid cells in extrarenal anatomic sites may be divided into specific tissue-based diagnostic categories, such as poorly differentiated neoplasms, including sarcomas, carcinomas and carcinosarcomas, and metastatic sarcomas within a preexisting carcinoma (6). Adenocarcinoma may also manifest various metaplastic features, including sarcomatoid dedifferentiation; this distinctive histological entity has been previously described as adenocarcinoma with sarcomatoid dedifferentiation, true carcinosarcoma, and poorly differentiated adenocarcinoma (6). Adenocarcinoma with rhabdoid features may exhibit similar morphological characteristics to those of malignant rhabdoid tumors, and therefore, the existence of malignant extrarenal rhabdoid tumors as a distinct clinicopathological entity remains open to discussion (12). The current study presents the 10th case of poorly differentiated adenocarcinoma with rhabdoid features arising in the colon and reviews the previously reported cases. To the best of our knowledge, this is the first case of colonic carcinoma with rhabdoid features coinciding with appendiceal mucinous cystadenoma. The study was approved by the ethics committee of Chosun University Hospital (Institutional review Board of Chosun university hospital, Gwangju, Korea), who waived the requirement for written informed consent due to the nature of the study.

## Case report

### Clinical summary

A 73-year-old male was admitted to the Department of Surgery, Chosun University Hospital (Gwangju, Korea) with a 2-week history of pain in the right lower quadrant. Abdominal computed tomography (CT) with enhancement by contrast media revealed acute appendicitis and a cecal edema; based upon this finding and inflammation, cancer was suspected. Upon a clinical diagnosis of acute appendicitis, appendectomy was performed. During surgery, a cecal mass was identified, and an examination of the frozen section of the cecal lesion revealed malignancy. Therefore, in addition to appendectomy, a right hemicolectomy with regional lymph node dissection was conducted.

### Pathological findings

**Figure 1:**
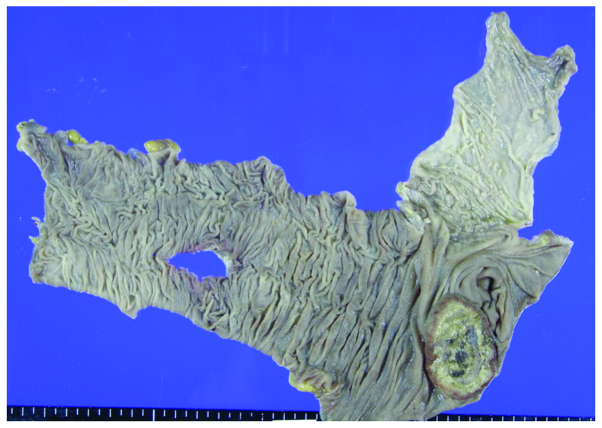
Protruding mass of 4.0×3.0×1.5 cm in size with central necrosis was identified in the cecum.

**Figure 2:**
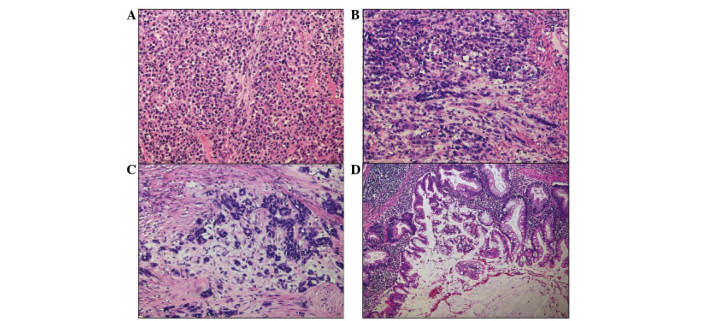
(A) Microscopically, the tumor was composed of loosely cohesive, rhabdoid cells. The characteristic strongly and homogeneously acidophilic cytoplasm of the rhabdoid tumor cells was observed with lateral displacement of the nuclei. (B) Transition from adenocarcinoma (lower) to rhabdoid areas (upper) was noted. (C) Small amount of adenocarcinoma component was identified and (D) appendiceal mucinous cystadenoma was identified.

**Figure 3:**
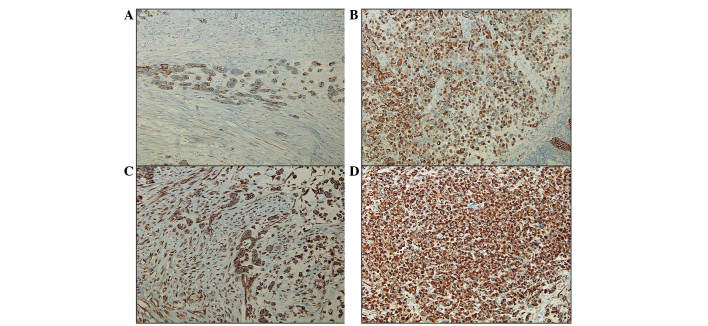
Immunohistochemically, the tumor cells of the adenocarcinoma component and rhabdoid component were positive for cytokeratin: (A) Adenocarcinoma component, (B) rhabdoid component; and positive for vimentin: (C) Adenocarcinoma component; (D) rhabdoid component.

**Figure 4:**
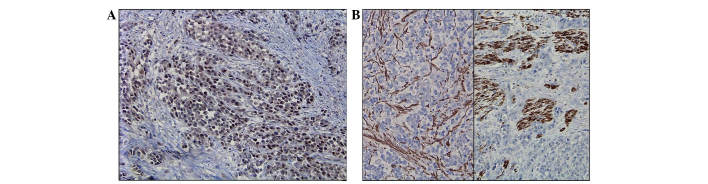
Immunohistochemically, the tumor cells were immunoreactive for (A) mutL homolog 1, but negative for (B) smooth muscle actin and (C) desmin.

A protruding mass of 4.0×3.0×1.5 cm in size, with central ulceration and necrosis was identified in the cecum (Fig 1). Microscopically, the tumor was composed of loosely cohesive, rhabdoid cells which grew in a diffuse, solid and focal alveolar pattern (Fig. 2A). Transition of the gland-forming adenocarcinoma to the area or malignancy demonstrating prominent rhabdoid features was identified (Fig. 2B); the amount of adenocarcinoma component forming the glandular structure was <1% of the total tumor area (Fig. 2C). The most noteworthy feature of these rhabdoid tumor cells was the strongly and homogeneously acidophilic cytoplasm of the tumor cells, with lateral displacement of the nuclei (Fig. 2A). Extensive necrosis was observed and regional lymph node metastasis was also identified in four out of 45 regional lymph nodes, pN2a. The metastatic lesion was entirely composed of rhabdoid tumor cells. Immunohistochemically, the tumor cells of the adenocarcinoma and rhabdoid components were positive for CK (Fig. 3A, adenocarcinoma component; Fig. 3B, rhabdoid component), VMT (Fig. 3C, adenocarcinoma component; Fig. 3D, rhabdoid component) and MLH-1 (Fig. 4A), but negative for skeletal muscle marker, desmin and smooth muscle actin (Fig. 4B and C). In addition to the malignant tumor, separated appendiceal mucinous cystadenoma was also identified (Fig. 2D). The final diagnosis was poorly differentiated adenocarcinoma with prominent rhabdoid features, combined with appendiceal mucinous cystadenoma. At two months following surgery the patient succumbed to peritoneal seeding and metastasis of liver and bone.

## Discussion

RT was originally described as a primary renal neoplasm (13), however, examples of a morphologically similar neoplasms have been subsequently identified in a number of other sites, including soft tissues (14). Of these, RTs of the colon are extremely rare, and to the best of our knowledge, only nine cases have been previously reported in the English language literature (2–9). Histologically, RT is characterized by the unique morphological feature of proliferating rhabdoid cells, which have an abnormally located large nucleus and prominent nucleoli, and a typical eosinophilic inclusion of aggregated intermediate filament (12,15). Only two types of RT have been reported: One is the pure type and the other is described as the composite type (7). Chetty *et al* (8) proposed that, in the composite type of RT showing malignant rhabdoid cells coexisting with adenocarcinoma, the rhabdoid cells may have been derived from sarcomatoid dedifferentiation of malignant epithelial cells. This is in contrast to the pure type, where the tumors are composed exclusively of rhabdoid cells, without any other epithelial component. In total, five of the nine cases previously reported were composite type, and four cases were pure type (2–9). The present case was determined to be composite type RT, concurrent with mucinous adenoma of appendix. A marginal volume of gland-forming adenocarcinoma was identified, which accounted for <1% of the total tumor volume. Transition of adenocarcinoma component to the rhabdoid area was also observed.

The previously reported cases were diagnosed as malignant extrarenal rhabdoid tumors (MERTs) or adenocarcinoma with rhabdoid features. Although MERTs have been well-defined and characterized as a clinicopathological entity (ICD-O 8963/3) (16), the existence of a composite carcinoma consisting of an epithelial component associated with rhabdoid cells indicates that rhabdoid cells may be the result of sarcomatous dedifferentiation (12). Furthermore, the rhabdoid cells of the pure and composite forms of RT exhibit similar morphological and immunohistochemical characteristics. This feature also suggest that rhabdoid cells may have dedifferentiated from epithelial tumor cells, and not from metastatic sarcoma or metastatic malignant renal rhabdoid tumors (6).

All rhabdoid colorectal tumors (RCTs), including those previously reported and the present case, are listed in Table I (2–9). All RCTs have similar clinicopathological features. The majority of MERT cases affect infants, whereas RCTs exclusively affect elderly patients; the mean age at diagnosis was 73.5 years. The predominant site involved was the cecum, six out of 10 cases; the other sites of involvement were the transverse colon, two out of 10 cases; sigmoid colon, one out of 10 cases; rectum, one out of 10 cases. No gender predilection was evident. (male:female ratio, 6:4). In total, eight out of 10 cases exhibited regional lymph node metastasis at diagnosis, and four out of 10 cases showed hepatic metastasis. The biological behavior was very aggressive; seven patients succumbed to the disease within eight month (mean, eight months and two weeks). The patient in the present case succumbed to the disease two months following surgery.

Several genetic abnormalities were reported in previously published cases. Kono *et al* (6) reported a case of cecal adenocarcinoma, which showed prominent rhabdoid features on immunohistochemical, ultrastructural and molecular analyses, and the authors observed strong expression of human mutL homolog 1 (hMLH1) protein in the nuclei of the rhabdoid cells. However, microsatellite instability (MSI) at five polymorphic markers (BAT25, BAT26, D2S123, D5S346, D17S250) was not observed in the rhabdoid cells. Pancione *et al* (2) reported a novel case of colon rhabdoid carcinoma associated with a positive CpG island methylator phenotype and BRAF mutation. The authors revealed that the promoter regions of four out of five specific genes that define the CpG island methylator phenotype, including MLH1, were methylated. Additionally, MSI was detected. Furthermore, a mutation in BRAF V600E was detected, however, no KRAS mutation was identified. This indicated that genetic and epigenetic events may be involved in the occurrence and progression of this rare and aggressive phenotype, revealing a potential implication for its management. In the study by Remo *et al* (7), all neoplastic cells were observed to express hMSH2 protein but were negative for hMLH1; a BRAF V600E mutation was identified, but no KRAS mutation was present, which is consistent with the study by Pancione *et al*. Remo *et al* (7) also reported that the promoter regions of the characteristic subset of genes for the CIMP status (NEUROG1, IGF2, RUNX3, SOCS1, including MLH1) were hypermethylated, suggesting the presence of a CIMP+ and MSI-high tumor. In the present case, tumor cells were immunoreactive for MLH1, which indicates that a similar genetic event may have occurred, causing this abnormality.

In conclusion, the current study reports the 10th case of RCT (composite type) with a review of the previously reported RCTs. In the present case, separate appendiceal mucinous cystadenoma was concomitant with RCT. All RCT cases exhibit similar clinicopathological features, as well as the characteristic histological feature of sarcomatous dedifferentiation of rhabdoid cells, which appears to indicate aggressive biological behavior. Further investigations into this highly aggressive colonic carcinoma showing rhabdoid feature are required in order to determine specific and effective treatment for this tumor type.

## Figures and Tables

**Table I:** Reported cases of colorectal tumor with prominent rhabdoid feature.

Author	Age/gender	Site	Size, cm	Histology	LN metastasis	Outcome	Other
Chetty *et al* (8)	72/F	Cecum	6×5	Composite	+	STD (3 mo)	None
Yang *et al* (3)	75/M	Transverse	10×10	Pure	+	STD (2 wk)	None
Marcus *et al* (4)	84/F	Transverse	7×6	Pure	−	Alive (12 mo)	None
Nakamura *et al* (5)	76/M	Cecum	14×8	Pure	+	STD (12 wk)	None
Kono *et al* (6)	66/M	Cecum	13×13	Composite	+	STD (6 wk)	None
Pancione *et al* (2)	71/M	Cecum	10×10	Pure	−	STD (8 mo)	None
Remo *et al* (7)	73/F	Cecum	10×8	Composite	+	STD (6 mo)	PC
Lee *et al* (9)	62/M	Sigmoid	4.5×4.0	Composite	+	Alive (36 mo)	None
	83/F	Rectum	6.5×4.3	Composite	+	STD (1 mo)	None
Present case	73/M	Cecum	4×3	Composite	+	Alive (4 wk)	Adenoma

LN, lymph node; F, female; M, male; STD, succumbed to disease; mo, month; wk, week; PC, polyposis coli.
